# Evaluation of Volatile Organic Compounds from Spotted Lanternfly (*Lycorma delicatula*) Eggs Using Headspace Odor Sampling Methods

**DOI:** 10.3390/insects15100739

**Published:** 2024-09-26

**Authors:** Ariela Cantu, Edgar O. Aviles-Rosa, Nathaniel J. Hall, Paola A. Prada-Tiedemann

**Affiliations:** 1 Forensic Analytical Chemistry and Odor Profiling Laboratory, Department of Environmental Toxicology, Texas Tech University, Box 41163, Lubbock, TX 79416, USA; 2 Canine Olfaction Laboratory, Animal and Food Sciences Department, Texas Tech University, Box 42141, Lubbock, TX 79409, USA

**Keywords:** spotted lanternfly, *Lycorma delicatula*, odor profile, canine detection, solid-phase micro-extraction (SPME), gas chromatography-mass spectrometry (GC/MS)

## Abstract

**Simple Summary:**

National biosecurity efforts provide a set of precautions that aim to prevent the introduction and spread of harmful organisms, including non-native tree pests such as insects and disease-causing organisms. The spotted lanternfly is an example of a current biosecurity threat, which, while native to China, was discovered in US territory in 2014. Canine detection is an emerging tool in this pest detection effort; however, an important aspect of further eradication developments is to understand the odor chemistry of this insect. The goal of this study is to evaluate the headspace of lanternfly eggs via the instrumental approach of solid-phase microextraction with gas chromatograph-mass spectrometry (SPME-GC/MS). This study presents samplings of cold-killed spotted lanternfly eggs and relevant distractor samples to compare odor profiles. The developed method is a feasible approach for VOC analysis. The results indicate that lanternfly egg and cricket samples share a common VOC, dodecane, while the lanternfly egg and bark samples share anisole as a common odor volatile. This study provides an initial snapshot of the VOC makeup of *L. delicatula*, as well as implications for long-term storage, that can aid in the development of a canine training aid for early detection and mitigation strategies.

**Abstract:**

The spotted lanternfly (SLF) is an invasive species native to China. It was first discovered in the United States in Pennsylvania in 2014. It is known to cause great economic damage by destroying various crops, specifically grape vines, and therefore, several efforts have been made to control and mitigate its spread from the Northeast. Canine detection is a useful detection tool; however, it is crucial to understand the volatile organic compounds emitting by this pest to better direct canine training paradigms to prevent false alerts and to understand potential volatile markers of importance indicative of this species. The purpose of this study is to address the gap in research regarding the volatile organic compound (VOC) profile of SLF to better inform pest control mitigation strategies. Instrumental analysis was performed utilizing SPME-GC/MS on cold-killed SLF eggs, dried crickets, and tree bark. Differences in detected VOCs within each sample set depicted distinctive odor profiles for each matrix tested. Storage of these samples also depicted VOC accumulation variation as a function of time, thereby providing implications for long-term storage and sample handling for these types of training aids in canine applications.

## 1. Introduction

*Lycorma delicatula*, more commonly known as the spotted lanternfly (SLF), is an invasive species from the Hemiptera: Fulgoridae family [1]. This pest is native to China and was first labeled as a native pest when it was discovered in South Korea in 2004 [2]. There are numerous reports of the pest wreaking havoc on apple trees, stone fruit, and especially grape vines in South Korea [3]. When it was discovered for the first time in Pennsylvania in the United States, there were many concerns about the possible economic damage it could cause [4]. Since its discovery in the United States in 2014, it has spread across several states in the Northeast, and efforts have been made to control its spread due to fears of environmental and economic damage.

The spotted lanternfly is considered an univoltine species, which means that it only reproduces once a year, with egg-laying occurring from August through early November [5]. A process called overwintering occurs after the eggs are laid, where the eggs remain in their oothecae during the winter until they begin to hatch in late spring [6]. Their lifecycle consists of a total of 4 instars, starting when the eggs hatch in late spring and ending when they become adults by late summer. Eggs are usually laid on its preferential host plant *Ailanthus altissima* (*Miller*) *Swingle*, better known as the tree of heaven, but it is also known to feed on over 70 other plant and fruit species [7]. When *L. delicatula* feed heavily in swarms, they can cause honeydew to leak onto the plant, which can lead to sooty mold growth and, as a result, can cause the plant to be blocked from sunlight, hindering photosynthesis [8]. Pennsylvania vineyards have reported SLF infestations that resulted in up to a 90% crop yield loss. They also reported that vines in areas where SLF heavily fed did not survive the winter, and vines that did survive did not bear any fruit in the subsequent year [9]. This destruction of plants and fruits raises a biosecurity threat concern as to the damaging effects *L. delicatula* can have. Egg masses can be laid on numerous things, such as cars, shipping pallets, and rail cars, which can be useful sources to promote the spread of the pest to other areas [10]. The use of detection dogs for biosecurity screening at various borders and entries could be a useful tool to try and limit the spread of invasive species, such as in the case of *L. delicatula* [11,12,13,14].

Currently, most research surrounding SLF involves the evaluation of pest control measures such as traps, pesticides, or various mitigation efforts. Sticky band traps are used by placing the sticky band around the trunk of the tree and capturing SLF as they travel up and down the trees [5]. Some obvious limitations with sticky band traps are that they can only be used on trees, as the band must wrap around the trunk, so their use with smaller plants that SLF invade would be more difficult. Another issue seen with sticky band traps is the possibility of trapping non-target insects or even other small animals, such as birds, in the traps, which would obviously not serve the proper purpose of the traps [5]. In a study conducted by Franceses et al. in 2019, they worked on developing a trapping method by observing the various behaviors of *L. delicatula* [6]. They witnessed nymphs climbing the trunk of their host plant to try and reach the top, a particular behavior unique to *L. delicatula* [6]. Most of the traps currently used capture the nymphs as they climb and fall off their host plant; however, there are several problems seen with these kinds of traps. Some of these issues include the learned behavior of *L. delicatula*, as the traps generally work on the younger first- and second-instar nymphs; however, the older nymphs and adults learn to either completely avoid the glue traps, or they are able to pull themselves out of the traps all together [6]. During this study, five different types of traps were evaluated on their ability to successfully trap *L. delicatula* while avoiding trapping any non-target insects or debris. Taking all the information collected from their observations, they were able to determine which kinds of traps worked best and what improvements could be made to already existing traps to increase their success rates. In a similar fashion, a study conducted by Cooperband et al. in 2018 aimed to determine if chipping could be used as a successful mitigation strategy against *L. delicatula* [7]. Their results found that there were no nymphs present in the barrels with the chipped tree material, while there were about 500 nymphs present in the control barrels with the tree material intact [7]. Based on the results of this study, researchers were able to determine that chipping the host tree *A. altissima* during *L. delicatula*’s overwintering season would be a useful mitigation strategy against this pest [7]. Other preventative methods that have been researched include the use of various insecticides [8], as well as an innate egg parasitoid [9] to help control population numbers. Previous studies have employed similar methods to determine the odor profiles of various types of insects, including larvae, pupae, flour beetles, and maggots [10,11,15].

Biosecurity detection dogs have been trained to detect invasive insects and pests before; for example, in a study by Moser et al. in 2020, detector dogs were trained using scent extracts and dead samples of *Musgraveia sulciventris* (*AFD*) [4]. The results from this study found that odor extracts could be used as a training aid for detector dogs as a substitute for live samples, with the dogs still able to be successfully trained in identifying their target odor [4]. Another study was performed to determine if dogs could be trained to detect red imported fire ants, *Solenopsis invicta* (*Buren*), as well as locate the ants’ nest in a field setting [16]. It was determined that the dogs could not only detect the fire ants, but they could also discriminate it from three other species, as well as locate the nests [16].

Dogs have also previously been trained to detect bark beetle-infested trees using a pheromone component from *Ips typographus* (*Linnaeus*) [17]. The study found that dogs could be trained to detect and locate infected bark from 100 m or more, which showcases the diverse use of detector dogs [17]. In a similar study conducted by Essler et al. in 2021, tests were conducted to determine if detector dogs could be trained with dead *L. delicatula* egg masses and if this identification could then be transferred to detecting live egg masses [12]. The study found that the dead *L. delicatula* eggs could be used as a potential training aid for the detector dogs; however, while these studies were also performed in a lab, there was no chemical odor profile evaluation on the utilized samples [12]. An important suggestion for future study in mitigation strategies using canine detection teams is the importance of testing alternate insects’ odor volatiles to better narrow the target odor source. This work was continued in a similar study by Aviles-Rosa et al. [14], who attempted to train detection dogs to alert to cold-killed SLF masses. Several distractor odors were used, such as crickets, red maple tree bark, grasshoppers, and tree of heaven bark. This was to ensure that the dogs were trained on the scent of the cold-killed SLF egg masses and not other insects. In this study, it was determined that detection dogs could be a valuable tool in the detection of SLF eggs, as the dogs used in their study had specificity and sensitivity rates greater than 95% [14].

Various mitigation and prevention techniques have been developed to deal with the spread of SLF, using canines as biological detectors for this invasive species as an up-and-coming technique for pest management efforts. While detection dog efforts are being developed and refined, a constant gap in the research surrounding SLF detection via canine means remains, and that lies in the chemical understanding of the odor profile and in identifying volatile markers for this targeted species that can be used to facilitate detection dog training. This study addressed this issue and helped provide foundational information on the chemical odor signature of SLF to better inform pest control mitigation strategies utilizing trap technologies and canine detection teams. The objectives of this research were to (1) characterize the chemical odor profile of cold-killed SLF eggs, (2) compare chemical odor signatures to other environmentally relevant distractor samples, and (3) monitor the persistence of these chemical odor signatures over two different 6-week sampling periods, one under frozen conditions and another under ambient dissipation conditions (open air vs. closed) for storage implications.

## 2. Materials and Methods

To perform a chemical characterization of the odor volatiles emitted from the spotted lanternfly (SLF) eggs and associated samples under study, instrumental analysis was performed utilizing solid-phase microextraction gas chromatography-mass spectrometry. SLF egg masses were collected in Winchester, VA, in 2023. They were scraped from the bark of the tree and frozen at −80 °C for 72 h. Samples were shipped to Texas Tech University and stored at −20 °C until used for extraction. For comparative headspace odor evaluation, additional samples included crickets and tree bark to monitor other environmentally relevant odors from surrounding SLF collection areas. The tree bark (Callery pear) used for analysis was from the same tree from which the eggs were collected. The bark samples were received with no prior information on the number of trees sampled, the tree’s exact location within the field, or the environmental conditions at collection. All samples were stored in 7 mL glass vials with a screw cap and PTFE/silicone septa (Supelco, Sigma Aldrich, Burlington, MA, USA) for SPME extraction procedures. Metal tweezers and a spatula were used to transfer the samples from the mason jar they were received in into the 7 mL headspace extraction vials. Sample weights are summarized in Table 1. All replicate samples were divided into approximately equal weights from the original sample weight received to perform the analytical evaluation. To control for any volatile odor contamination, all sampling glass vials were pretreated prior to use. This procedure entailed an initial 3 h bake at 105 °C on all glass vials and a quick 15 min bake at the same temperature for the lids, septa, and spatula tool.

### 2.1. Frozen Storage Study

SLF egg samples were received in a mason jar; however, for analysis purposes, all samples were transferred to pretreated 7 mL glass vials with a sterilized spatula and distributed by weight into six replicates for headspace odor sampling. A total of six replicates were also prepared for the cricket samples (Fluker’s Cricket Farm, Inc., Port Allen, LA, USA). Prior to injection, each vial was weighed and labeled to monitor sample weight distribution, as shown in Table 1.

**Figure 1:**
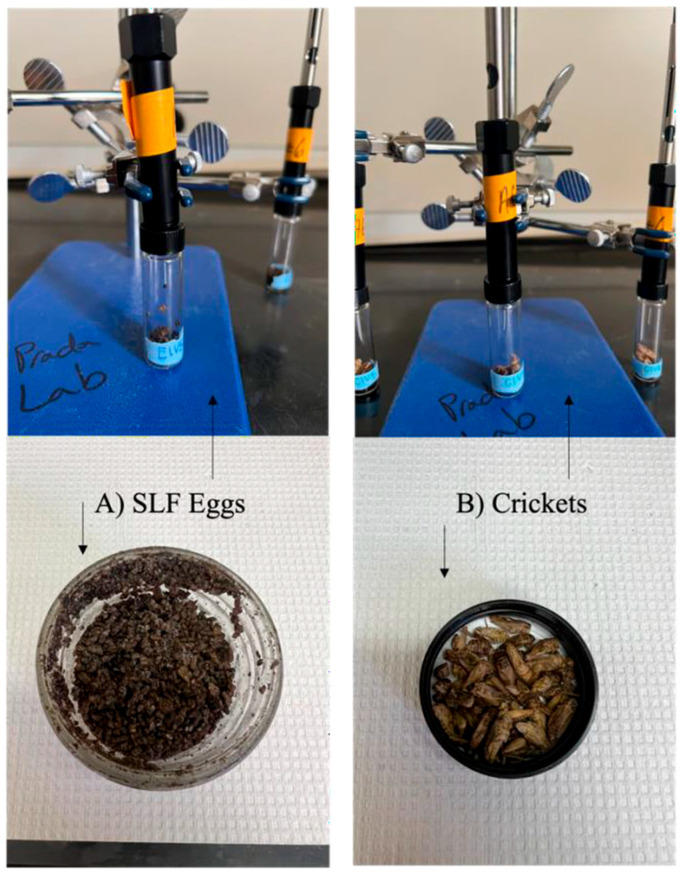
Sample vials containing (**A**) SLF eggs and (**B**) crickets with SPME fiber injected in the headspace.

Prior to any extraction procedure, both SLF eggs and related insect samples were thawed at laboratory room conditions for 1 h. Once this 1 h thaw was over, each vial was injected with the SPME fiber, where the fiber was exposed to the headspace of the SLF eggs and cricket samples for 24 h at standard laboratory conditions (see Figure 1). The temperature and humidity were recorded at every injection time. The average temperature during the 6-week extraction time was 18.0 °C, and the average humidity was 27%. After the 24 h headspace extraction, the SPME fiber was manually injected into the GC injection port for GC-MS analysis. Once the 24 h headspace extraction was finished, the samples were put back into the freezer (−20 °C) until they were taken out for analysis on a weekly basis for six weeks. The SLF eggs and cricket samples were re-sampled once a week for 6 consecutive weeks to analyze the stability of the VOC odor profile for each specimen over time.

### 2.2. Longevity: Effect of Ambient Storage Study

In this study, a new set of SLF egg and cricket samples were analyzed in a similar way; however, the samples were not frozen between sampling times, and there were two separate sets of samples analyzed. There was one set of SLF egg and cricket samples that were left in laboratory conditions between sampling times with their vial lids on, and there was a second set of SLF egg and cricket samples left in laboratory conditions between sampling times with their vial lids left off. The only time this second set had their vial lids on was during the 24 h headspace extraction time. The extraction methodology was the same for the longevity frozen study, with the samples being evenly distributed into 7 mL vials, six replicates of each. Prior to the initial injection, each vial was weighed and labeled to monitor sample weight distribution, as shown in Table 2.

In Table 2, the eggs and crickets in open vials indicate the samples that were left with their vial tops off between extraction periods, and the eggs and crickets in closed vials indicate the samples that were left with their vial tops on between extraction periods. The only times the samples were in freezer conditions was prior to sampling procedures. Prior to week 0’s extraction time, the samples were removed from the freezer and allowed to thaw for 1 h before SPME extraction procedures. Once this initial 1 h thaw was over, each vial was injected with an SPME fiber where the fiber was exposed to the headspace of the SLF eggs and cricket samples for 24 h at standard laboratory conditions. The temperature and humidity were recorded at every extraction time, and the average temperature during the 6-week extraction was 18.6 °C, and the average humidity was 57%. The open and closed SLF egg and cricket samples were re-sampled weekly for 6 consecutive weeks to monitor storage temperature conditions on the detected VOC profile of each specimen over time. Once the samples were finished with their 24 h headspace extraction, the samples were left on a benchtop in the laboratory until they were analyzed the following week.

### 2.3. Additional Distractor Odor Samples

As previously mentioned, other specimens were analyzed as environmentally relevant distractor odors innate to the environment of SLF. Due to logistical issues with sample acquisition, the other distractor odor analyzed, bark from the Callery pear, was not included in either shelf life storage study. To provide a snapshot of the volatile odor profile of the bark samples, a total of 12 (*n* = 12) pieces were prepared for injection with the same method as the other specimens. Once the samples were received, they were transferred from the mason jar into 12 separate 7 mL vials. The weights of the bark samples can be seen in Table 3.

### 2.4. Instrumentation Procedures (SPME-GC/MS)

To extract the VOCs from each specimen, solid phase microextraction (SPME) was used. The SPME fiber utilized in this study was a 50/30 μm polydimethylsiloxane/divinylbenzene (PDMS/DVB) SPME fiber (Supelco, Sigma Aldrich, St. Louis, MO, USA). Each SPME fiber was conditioned for 30 min at 250 °C at least three times to guarantee each fiber was clean and ready to be used prior to specimen sampling. A blank fiber instrument run was then performed to ensure no contaminants or lingering volatiles remained on the fiber.

An Agilent Technologies GC 7890A model with an Agilent Technologies 5975C inert XL MSD with triple-axis detector (Agilent Technologies, Santa Clara, CA, USA) was used for the separation and analysis of the compounds extracted by the employed SPME fiber (33). An Rtx^®^-5 capillary 30 m × 250 μm × 0.25 μm column (Restek Corporation, Bellefonte, PA, USA) was used. Before analyzing the samples, an instrument blank was run on the GC-MS instrument to check its performance with blank vials for calibration purposes. This also validated that the instrument was running correctly prior to sample analyses. Ultra-high purity helium was used as a carrier gas at a flow rate of 1.0 mL/min. Samples were injected in the splitless mode. The oven temperature started at 35 °C for 5 min, increased to 240 °C at a rate of 10 °C/min then held again for 4 min. The total run time for analysis was 29.5 min. Mass spectra were repeatedly scanned from 45 to 550 amu. Compounds were identified using the NIST 17 (2017, Version 2.3) mass spectral reference library and verified with external standard chemical calibration where possible (anisole and dodecane). The criteria for the compounds identified were those with detected peaks greater than or equal to a mass spectral match quality of 90% or above.

### 2.5. Data Analysis

All generated data were analyzed using Chemstation software version 10.1.49 (Agilent Technologies, Santa Clara, CA, USA) and the National Institute of Standards and Technology mass spectral library (NIST 2017) for compound identification. Compounds known to be products of the column or sampling process were not included in the analysis. Principal component analysis (PCA) was used to compare the variances in the detected odor profiles from each specimen across targeted weeks of analysis by using 2D scatter plot graphing. Further statistical analysis focused on performing a one-way ANOVA analysis to evaluate the effect of storage on VOC dissipation. Statistical analysis was performed using JMP Pro 16.0.0, SAS Institute Inc. 2021 (Cary, NC, USA). The significance level for the ANOVA analyses was *p* < 0.05.

## 3. Results

### 3.1. Chemical Characterization of SLF Eggs, Crickets, and Bark

**Figure 2:**
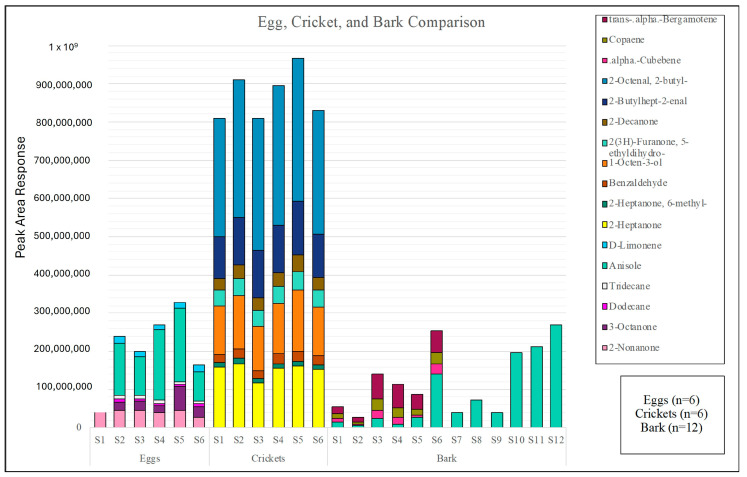
Stacked bar graph representing the relative peak area response of the odor profiles of the SLF eggs, crickets, and bark upon initial sampling, day zero.

A volatile odor characterization of SLF eggs, crickets, and bark was conducted. A comparison of the overall abundance of volatiles present in each sample can be seen in Figure 2, depicted in a color odor chart highlighting the primary odor volatile array for each specimen at day zero. The crickets yielded the highest VOC abundance, approximately 2.10 × 10^7^, while the bark and eggs yielded low abundance at or below 5.00 × 10^6^. Comparing the two insect sample matrices, it can be observed that the crickets had 4.2 times the volatile abundance compared to the SLF eggs. These compounds are coined by the authors as primary odor volatiles, as they were present in all six of the SLF eggs and cricket samples, while the primary odor volatiles in the bark were present in at least six of the twelve samples. This chemical characterization consisted of SLF egg samples (*n* = 6), crickets (*n* = 6), and bark (*n* = 12). A total of six compounds were detected in the eggs: 2-nonanone, 3-octanone, dodecane, tridecane, anisole, and D-limonene. A total of eight compounds were detected in the crickets: 2-heptanone, 6-methyl-2-heptanone, benzaldehyde, 1-octen-3-ol, 5-ethyldihydro-2(3H)-furanone, 2-decanone, 2-butylhept-2-enal, and 2-butyl-2-octenal. A total of four compounds were detected in the bark samples: anisole, α-cubebene, copaene, and trans-α-bergamotene.

**Figure 3:**
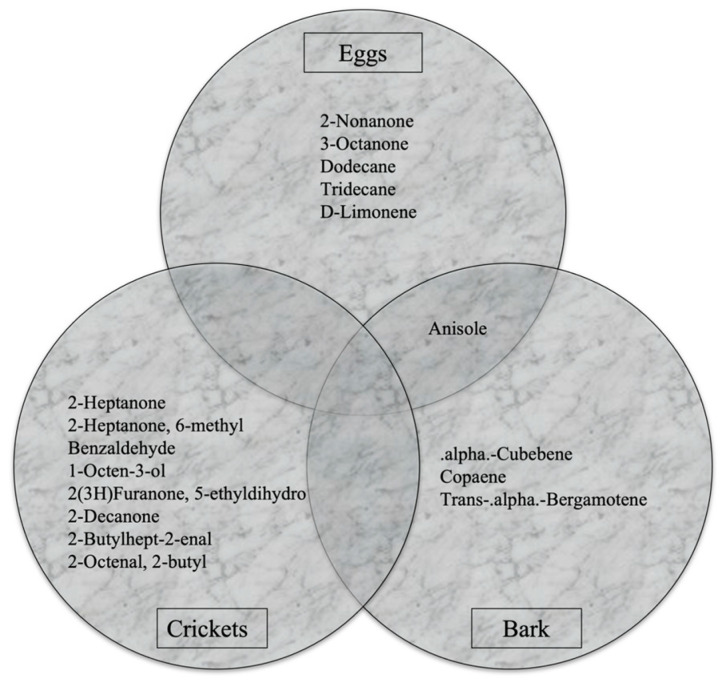
Venn diagram listing the primary and shared compounds from the SLF eggs, crickets, and bark.

Figure 3 shows a Venn diagram of the primary compounds from the SLF eggs, crickets, and bark. This figure is used to show the comparison of the compounds from each specimen with each other, and it shows that there was only one compound shared between any of the three specimens. The shared compound is anisole, and it is shared between the SLF eggs and the bark. As can be seen in Figure 2, anisole was detected in all six of the egg samples as well as all twelve of the bark samples.

### 3.2. Frozen Storage

Given the need for these samples to serve as canine training aids, an important variable to consider is the odor persistence of detected volatiles under storage conditions. Hence, a chemical characterization of the SLF eggs and crickets under frozen conditions was made over a 6 week period to monitor changes to the detected odor profile.

**Figure 4:**
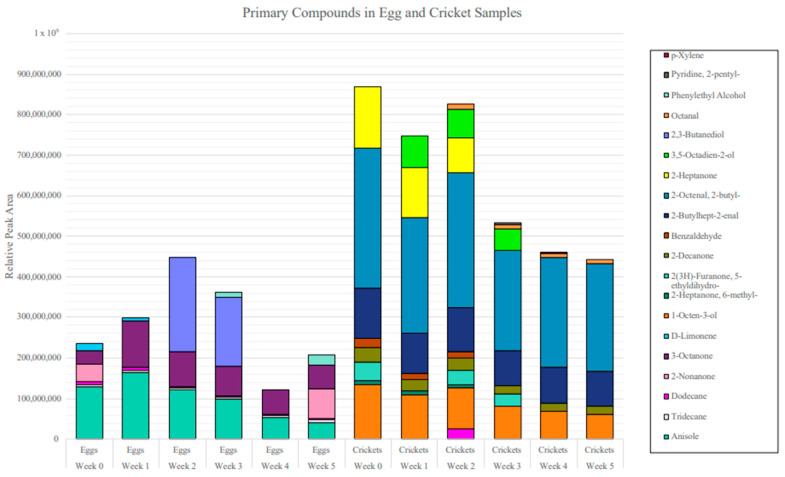
Stacked bar graph representing the relative peak area response of the odor profiles of the SLF eggs and crickets over a 6-week sampling period.

Figure 4 depicts a colored odor chart highlighting the primary odor volatile (i.e., odors appearing in all replicates) array for each specimen across the 6-week study period. The crickets depicted a more abundant odor profile measured by peak area response overall from week 0 to week 5. The highest relative peak area in the crickets was seen in week 0, with the value reaching 869,768,225, and the lowest relative peak area was seen in week 5, with the value reaching 441,360,932. A total of thirteen primary compounds were detected in the cricket samples at the different sampling times: dodecane, 1-octen-3-ol, 6-methyl-2-heptanone, 5-ethyldihydro-2(3H)-Furanone, 2-decanone, benzaldehyde, 2-butylhept-2-enal, 2-butyl-2-octenal, 2-Heptanone, 3,5-Octadien-2-ol, octanal, 2-pentyl-pyridine, and p-xylene. Of these compounds, four were present in all 6 weeks: 1-octen-3-ol, 2-decanone, 2-butylhept-2-enal, and 2-butyl-2-octenal. Compared to the crickets, the SLF eggs had a much more limited odor profile overall from week 0 to week 5. The highest relative peak area in the SLF eggs was seen in week 2, with the value reaching 448,212,236, and the lowest relative peak area was seen in week 4, with the value reaching 121,828,007. A total of eight primary compounds were detected in the egg samples at different times: anisole, tridecane, dodecane, 2-nonanone, 3-octanone, D-limonene, 2,3-butanediol, and phenylethyl alcohol. Of these compounds, four were present in all 6 weeks: anisole, tridecane, dodecane, and 3-octanone. Dodecane was the only compound shared between the SLF eggs and crickets; however, it was more prevalent in the egg samples, as it was present in all 6 weeks, compared to the crickets, where it was present in 1 week.

**Figure 5:**
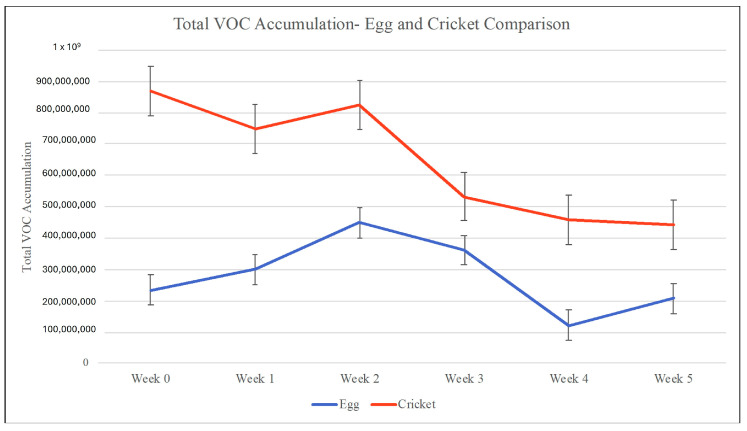
Total VOC accumulation graph from each specimen, SLF eggs and crickets, across the 6-week sampling period. Error bars indicate the standard error of the mean.

A total VOC accumulation from each specimen across the 6-week period can be seen in Figure 5. The average VOC accumulation for the SLF eggs was 278,706,213, and the average VOC accumulation for the crickets was 646,101,011. Comparing both insect samples, the crickets have more VOC accumulation across the board than the SLF eggs, with the greatest amount seen in week 0, with a value of 869,768,225. The greatest amount in the SLF eggs can be seen in week 2, with a value of 448,212,236. The VOC accumulation of both the SLF eggs and crickets stays within detection response the entire time, with the biggest decrease occurring in the eggs from week 3 to week 4.

**Figure 6:**
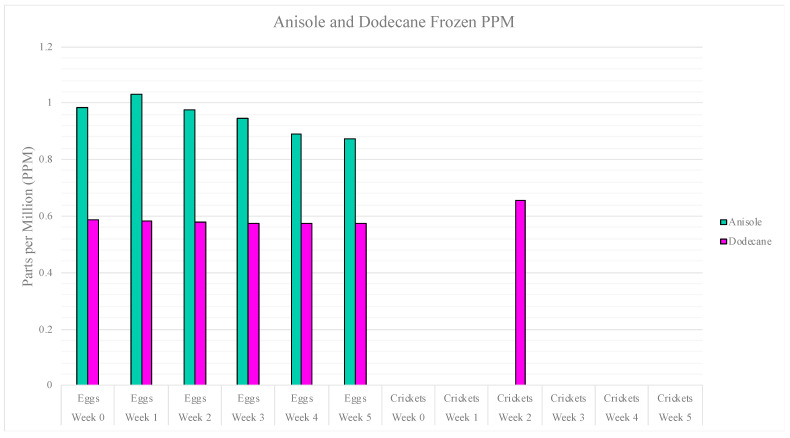
Bar graph representing the concentration of dodecane and anisole in the frozen storage study (PPM).

A calibration curve was created so the peak area response could be correlated to the amount of dodecane and anisole extracted across the egg and cricket samples in the frozen storage study. Using these two compounds, liquid solutions of various concentrations (5–100 ppm) were used to create external calibration curves for quantitation purposes. This was performed by injecting the range of liquid concentration solutions into the GC/MS system using the same method used for headspace sampling. An average response factor was then obtained and used to approximate the amount of VOCs being extracted by the SPME. These converted peak area responses to parts per million can be seen in Figure 6. As can be observed in the figure, anisole is present in a larger concentration across the egg samples, while dodecane is present in a larger concentration in the cricket samples.

**Figure 7:**
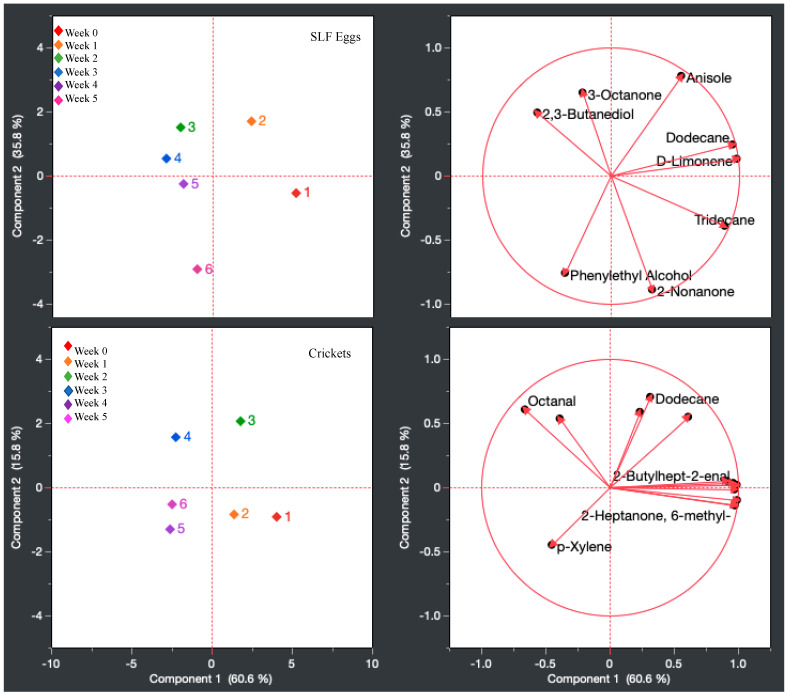
PCA of the primary compounds from the SLF eggs across 6 weeks (**top**) compared to the primary compounds from the crickets across 6 weeks (**bottom**).

To further exemplify the data acquired from the 6-week frozen sampling, a PCA statistical analysis was performed to monitor the odor profile changes throughout the tested period via clustering techniques. Figure 7 displays the PCA of the primary compounds from the SLF eggs and crickets across the 6-week study period. PCA multivariate analysis showed that the first principal component accounted for 44.4% of the variability in the data, while the second principal component accounted for 35.8% of the variability in the data in the eggs. Weeks 0 and 1 are grouped independently in their own quadrants, while weeks 2 and 4 are grouped together in the same quadrant, and weeks 4 and 5 are grouped together in the same quadrant. Samples from the earlier timeline (weeks 0–1) depict compounds such as anisole, dodecane, tridecane, and D-limonene as responsible for the diverging odor profile, while the later timeline samples (weeks 2–5) highlight 3-octanone, 2,3-butanediol, and phenylethyl alcohol as main drivers for the clustering during this period. For the crickets, PCA multivariate analysis showed that the first principal component accounted for 60.6% of variability in the data, while the second principal component accounted for 15.8%. Weeks 0 and 1 are grouped together in the same quadrant, while weeks 2 and 3 are grouped independently in their own quadrants. Weeks 4 and 5 are also grouped together in the same quadrant. For the earlier sampling period, the cricket samples had compounds such as 6-methyl-2-heptanone responsible for the clustering, while aldehydes such as octanal or aromatic hydrocarbons such as p-xylene are responsible for the advanced week odor profile clustering. It can be said that when comparing the SLF eggs and the crickets, dodecane is a compound that affects earlier sampling odor profiles more than the latter time periods of analysis.

### 3.3. Effect of Room Temperature Storage

The room temperature storage experiment focused on evaluating changes in the chemical odor profile as a function of storing samples in an open or closed vial at room temperature conditions. A characterization of the SLF eggs and crickets under room temperature conditions was made over a 6-week period.

**Figure 8:**
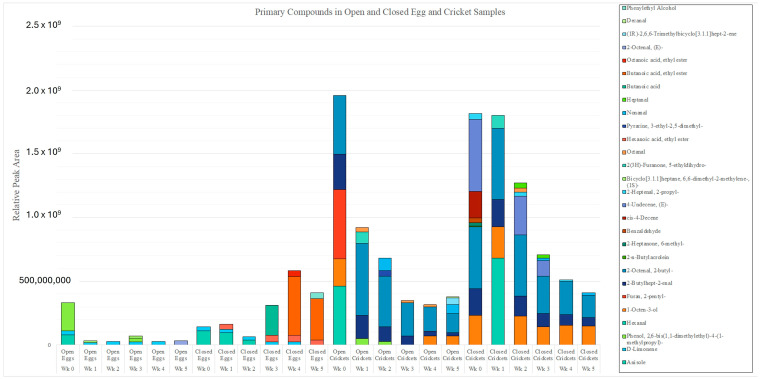
Stacked bar graph representing the relative peak area response of the odor profiles of the open and closed SLF eggs and crickets over a 6-week sampling period.

Figure 8 depicts a colored odor chart highlighting the primary odor volatile array for each specimen across the 6-week period. These compounds are coined by the authors as primary odor volatiles, as they were present in all six of the replicate samples per week for each specimen type. As can be observed in the figure, the crickets had a more expansive odor profile; however, around week 3, the abundance of the open crickets, closed eggs, and closed crickets began to even. The highest relative peak area in the open eggs can be seen in week 0, while the lowest relative peak area in the open eggs can be seen in week 2. The highest relative peak area in the closed eggs can be seen in week 4, while the lowest relative peak area in the closed eggs can be seen in week 2. The highest relative peak area in the open crickets can be seen in week 0, and the lowest relative peak area in the open crickets can be seen in week 4. Finally, the highest relative peak area in the closed crickets can be seen in week 0, while the lowest relative peak area can be seen in week 5. A total of 5 primary compounds were detected in the open egg samples: anisole, D-limonene, 2,6-bis(1,1-dimethylethyl)-4-(1-methylpropyl)-phenol, 6,6-dimethyl-2-methylene, (1S)-bicyclo[3.1.1]heptane, and (E)-2-octenal. There was one high-frequency compound among the open eggs, D-limonene, which was detected in 5 of the 6 weeks. The low-frequency compounds were anisole and (E)-2-octenal, which were detected in only 1 of the 6 weeks. A total of 12 primary compounds were detected in the open cricket samples: 2,6-bis(1,1-dimethylethyl)-4-(1-methylpropyl)-phenol, hexanal, 1-octen-3-ol, 2-pentyl-furan, 2-butylhept-2-enal, 2-butyl-2-octenal, 5-ethyldihydro-2(3H)-furanone, octanal, 3-ethyl-2,5-dimethyl-pyrazine, nonanal, (1R)-2,6,6-Trimethylbicyclo[3.1.1]hept-2-ene, and decanal. There were two high-frequency compounds among the open crickets, 2-butylhept-2-enal and 2-butyl-2-octenal, which were detected in all six weeks of sampling. A total of seven primary compounds were detected in the closed egg samples: anisole, D-limonene, ethyl hexanoate, butanoic acid, ethyl butanoate, ethyl ester octanoic acid, and phenylethyl alcohol. There were two high-frequency compounds among the closed eggs: D-limonene, which was detected in five of the six weeks, and ethyl hexanoate, which was detected in four of the six weeks. A total of 14 primary compounds were detected in the closed cricket samples: hexanal, 1-octen-3-ol, 2-butylhept-2-enal, 2-butyl-2-octenal, 2-n-butylacrolein, 6-methyl-2-heptanone, benzaldehyde, cis-4-decene, (E)-4-undecene, 2-propyl-2-heptenal, 5-ethyldihydro-2(3H)-furanone, octanal, nonanal, and heptanal. There were three high-frequency compounds among the closed crickets, 1-octen-3-ol, 2-butylhept-2-enal, and 2-butyl-2-octenal, which were detected in all six weeks of sampling. The low-frequency compounds were hexanal, 2-n-butylacrolein, 6-methyl-2-heptanone, benzaldehyde, cis-4-decene, 5-ethyldihydro-2(3H)-furanone, and octanal, which were detected in only one out of the six weeks of sampling. Only one compound was shared between any of the open/closed eggs and the open/closed crickets, 2,6-bis(1,1-dimethylethyl)-4-(1-methylpropyl)-phenol, which was present in the open egg and cricket samples. This compound was detected in the open egg samples in weeks 0 and 3 and the open cricket samples in weeks 1 and 2.

**Figure 9:**
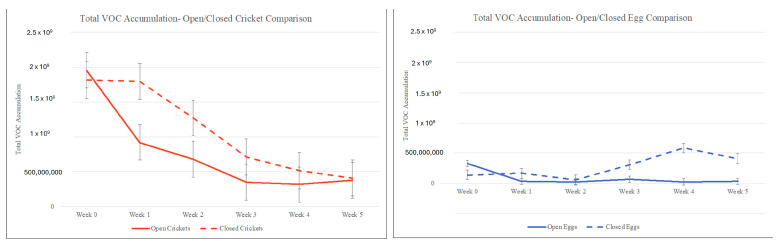
Total VOC accumulation graph from each specimen, open and closed SLF eggs and crickets, across the 6-week sampling period.

The total VOC accumulation from each specimen type across the 6-week period can be seen in Figure 9. The average VOC accumulation for the open SLF eggs was 87,859,910.5, while the average VOC accumulation for the closed SLF eggs was 279,285,727. The average VOC accumulation for the open crickets was 767,272,590, while the average VOC accumulation for the closed crickets was 1,085,909,475. The crickets had more VOC accumulation than the SLF eggs; however, around week 3, the abundance of the open crickets, closed eggs, and closed crickets began to even out. The highest abundance in the open eggs reached a value of 331,574,630, and the lowest abundance in the open eggs reached a value of 26,943,406.7. The highest abundance in the closed eggs reached a value of 582,760,430, and the lowest abundance in the closed eggs reached a value of 63,931,942. The highest abundance in the open crickets reached a value of 1,953,981,346, and the lowest abundance in the open crickets reached a value of 318,078,001. Finally, the highest abundance in the closed crickets reached a value of 1,815,987,183, and the lowest abundance in the closed crickets reached a value of 408,531,145.

**Figure 10:**
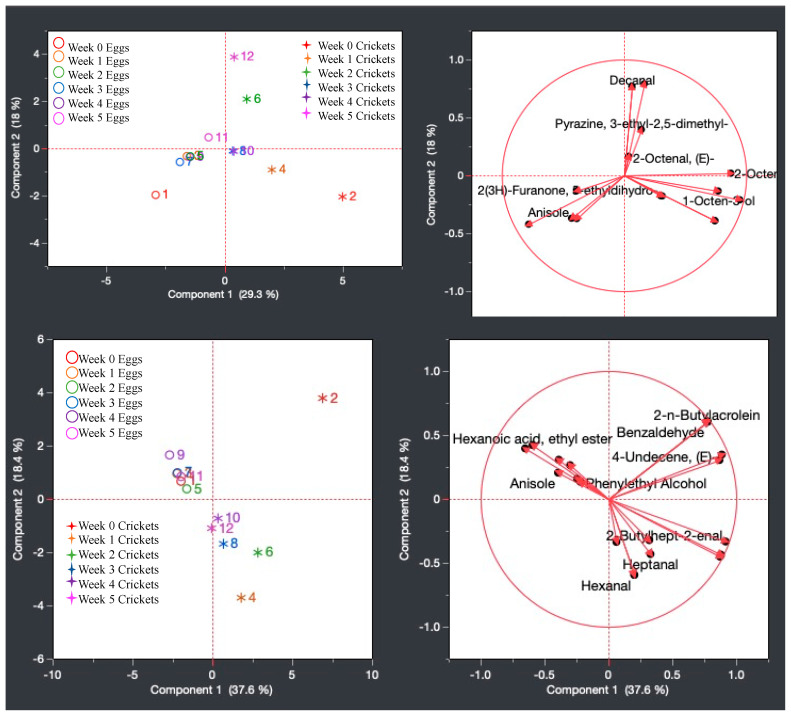
PCA of the primary compounds from the open egg and cricket samples across the 6 weeks (**top**) and the primary compounds from the closed egg and cricket samples (**bottom**).

A principal component analysis (PCA) was conducted to display the clustering components of the primary compounds from the open egg and cricket samples across the 6-week sampling period, as seen in Figure 10. PCA multivariate analysis showed that the first principal component accounted for 29.3% of the variability in the data, while the second principal component accounted for 18% of the variability in the data in the open samples. There is clustering seen in weeks 1 through 4 of the eggs, as well as in weeks 3 and 4 in the crickets. Week 5 of the eggs is grouped independently in its own quadrant, while week 0 is in the same quadrant as the cluster of weeks 1 through 4. In the crickets, weeks 0 and 1 are grouped in the same quadrant as the cluster of weeks 3 and 4, and weeks 2 and 5 are grouped in the same quadrant with each other. PCA multivariate analysis showed that the first principal component accounted for 37.6% of the variability in the data, while the second principal component accounted for 18.4% of the variability in the data in the closed samples. There is clustering seen in weeks 0 through 3, as well as week 5 in the egg samples. Week 4 in the eggs is not clustered, although it is in the same quadrant as the rest of the weeks. In the crickets, there is no clustering seen; however, weeks 1 through 5 are all grouped in the same quadrant, and weeks 4 and 5 are grouped very near each other. Week 0 in the crickets is grouped independently in its own quadrant.

**Figure 11:**
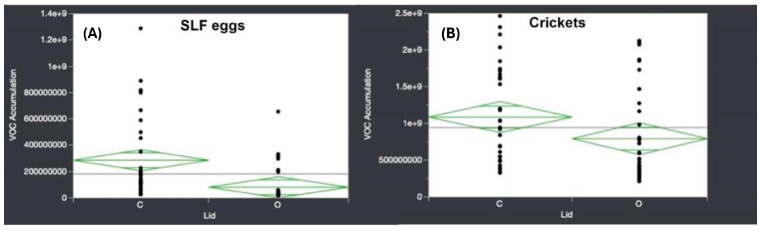
VOC accumulation distributions for (**A**) SLF eggs and (**B**) crickets as a function of sample lid condition (c—closed, o—open).

While the PCA depicted a lack of odor profile reproducibility over time for both lid conditions in the two insect species analyzed, an analysis of variance (ANOVA) was further conducted to statistically evaluate the effect of lid conditions on the overall volatile odor accumulation. ANOVA was used to test the significance of the primary compounds detected in this study as a function of specific targeted variables, in this case, sample lid condition. Figure 11 shows the distribution of the VOC accumulation based on lid sample condition (c—closed, o—open) for both SLF eggs and crickets. Setting the threshold for significance at *p* < 0.05, the SLF egg samples depicted a statistically significant effect at *p* = 0.0005, while the cricket samples yielded a non-significant effect at *p*= 0.0574. Figure 11 summarizes the distribution of VOC accumulation across both tested species.

## 4. Discussion

The objective of this study is to chemically characterize the VOC makeup of cold-killed spotted lanternfly eggs, as well as compare this profile to the VOC makeup of other distractor odors such as crickets and bark. Looking at the results from this initial characterization, a total of six primary compounds were detected in the eggs: 2-nonanone, 3-octanone, dodecane, tridecane, anisole, and D-limonene. A total of eight primary compounds were detected in the crickets: 2-heptanone, 6-methyl-2-heptanone, benzaldehyde, 1-octen-3-ol, 5-ethyldihydro-2(3H)-furanone, 2-decanone, 2-butylhept-2-enal, and 2-butyl-2-octenal. The difference in the odor profile of SLF eggs, crickets, and bark is a significant finding in this study. While other studies in the area of insect volatiles have targeted blowfly larvae and pupae, flower beetles, termites, maggots, eggs, larvae, and insect pupal residue, no studies have depicted the same set of compounds identified in this study, thereby suggesting a species-unique odor signature [10,11,15,18,19,20,21]. This indicates that a different odor profile is emitted depending on insect species, which indirectly corroborates the usefulness of canine detection by targeting a selected odor source for training purposes. This study supports the concept that small egg masses emit a detectable odor signature, and thus, their characteristic odor profile can be used as a viable target source that can be detected by dogs and analytical instruments. While instrumental odor availability cannot be linked to canine sensitivity, these results suggest that additional studies on different numbers of eggs encountered in infested sites may provide canine handlers valuable information as to the minimum amount of sample needed for optimal detection performance. Furthermore, testing SLF egg masses throughout different seasons and locations may further allow for understanding trends in odor profile as a function of insect feeding and breeding habitats.

A total of four compounds were detected in the bark samples: anisole, α-Cubebene, copaene, and trans-α-Bergamotene. Terpenes such as cubebene, copaene, and bergamotene correspond to plant-related materials, thereby corroborating the nature of the tested bark samples. Previous research has shown the presence of α-bergamotene in wild tobacco plants, indicating that this volatile is a moth-mediated pollination volatile as well as a chemical involved indirectly in plant defense mechanisms [22]. This study, therefore, corroborates previously reported chemicals innate to plant material that act as chemical signals for physical fitness and protection purposes.

The crickets depicted a wider array of compounds and abundance than the SLF eggs and bark. Only one compound was shared between all three specimen types, anisole, which was present in the SLF egg samples and the bark samples but not in crickets. In a study conducted by Yang et al., anisole was determined to be a potential environmentally friendly fumigant for postharvest pest control [23]. This could indicate that anisole is an innate part of the VOC profile of SLF eggs and the bark due to possible fumigation techniques in the environment, although further research should investigate fumigation chemicals and the effect on odor profiles generated to verify volatile transfer and origin.

Headspace solid-phase microextraction coupled with gas chromatography-mass spectrometry identified multiple consistently obtained compounds from the egg masses, indicating that it is a viable technique to utilize in determining the VOC profile of the spotted lanternfly, *Lycorma delicatula*. While the crickets exhibited a higher number and amount of compounds, it is important to note that the cricket was analyzed as a whole, therefore allowing a greater amount of surface area rather than just the egg. This discrepancy in the insect sample matrix could explain the almost 4× magnitude difference in volatile accumulation observed in the cricket when compared to the SLF egg. However, the technique demonstrates that a small matrix, such as an insect egg mass, can emit a distinctive chemical odor profile.

### 4.1. Frozen

The second objective of this study was to monitor the persistence of the chemical odor signatures of the SLF eggs and crickets over a 6-week longevity period under frozen conditions. A total of eight primary compounds were detected in the egg samples: anisole, tridecane, dodecane, 2-nonanone, 3-octanone, D-limonene, 2,3-butanediol, and phenylethyl alcohol. A total of thirteen primary compounds were detected in the cricket samples: dodecane, 1-octen-3-ol, 6-methyl-2-heptanone, 5-ethyldihydro-2(3H)-Furanone, 2-decanone, benzaldehyde, 2-butylhept-2-enal, 2-butyl-2-octenal, 2-heptanone, 3,5-octadien-2-ol, octanal, 2-pentyl-pyridine, and p-xylene. Dodecane is the only compound shared between the SLF eggs and crickets at one sample time point (week 2). This could indicate dodecane as a potential VOC marker for SLF eggs, and it has also been previously identified as an attractive compound in other pests, such as the grain beetle *Oryzaephilus surinamensis* [24]. Among the cricket samples, many compounds were found to be aldehydes, including benzaldehyde, octanal, 2-butylhept-2-enal, 2-butyl-2-octenal, and hexanal. Aldehydes are produced by insects, and butterflies and moths in the order Lepidoptera have been known to react to benzaldehyde and octanal [25]. Regarding the overall persistence of each specimen type, both the SLF eggs and crickets depicted a weekly change in the reported VOC profile. PCA analysis corroborated that the primary odor set of compounds did not cluster together as a function of the week, indicating continuous flux over time.

There was a drop in VOC accumulation in the SLF eggs from week 3 to week 4; however, the abundance went back up into week 5. This change could be a result of chemical degradation within the sample yielding new volatiles and/or changes in the abundance of original volatiles reported. The thaw/freeze cycle is also an important variable to consider, and future studies should evaluate the optimal thaw time to ensure odor availability is reached after the sample is frozen. The crickets had a higher VOC abundance overall, but neither specimen type had a complete depletion of odor accumulation over the sampling period. Thus, the ability of this method to detect odor volatiles even after 6 weeks of frozen storage suggests that cold storage safeguards volatile detection; however, it does not equate to volatile reproducibility. Other factors that could be explored in future studies include evaluating storage in amber vials to protect samples from light degradation and/or refrigerating at −80 °C conditions.

### 4.2. Room Temperature

The final objective of this study was to monitor the persistence of volatiles from the SLF eggs and crickets over a 6-week longevity period when stored in an open or closed vial at room temperature. A total of 5 primary compounds were detected in the open egg samples: anisole, D-limonene, 2,6-bis(1,1-dimethylethyl)-4-(1-methylpropyl)-phenol, 6,6-dimethyl-2-methylene-(1S)-bicyclo[3.1.1]heptane, and (E)-2-octenal. A total of 12 primary compounds were detected in the open cricket samples: 2,6-bis(1,1dimethylethyl)-4-(1-methylpropyl)-phenol, hexanal, 1-octen-3-ol, 2-pentyl-furan, 2-butylhept-2-enal, 2-butyl-2-octenal, 5-ethyldihydro-2(3H)-furanone, octanal, 3-ethyl-2,5-dimethyl-pyrazine, nonanal, (1R)-2,6,6-Trimethylbicyclo[3.1.1]hept-2-ene, and decanal. A total of seven primary compounds were detected in the closed egg samples: anisole, D-limonene, ethyl ester hexanoic acid, butanoic acid, ethyl ester butanoic acid, ethyl ester octanoic acid, and phenylethyl alcohol. A total of 14 primary compounds were detected in the closed cricket samples: hexanal, 1-octen-3-ol, 2-butylhept-2-enal, 2-butyl-2-octenal, 2-n-butylacrolein, 6-methyl-2-heptanone, benzaldehyde, cis-4-decene, (E)-4-undecene, 2-propyl-2-heptenal, 5-ethyldihydro-2(3H)-furanone, octanal, nonanal, and heptanal.

In both the SLF egg samples and the cricket samples, primary compounds were detected that were not present in the frozen study. This is most likely because half of the samples were left open, and these compounds could have come from the surrounding environment and are not an accurate representation of the VOC profile of either the SLF eggs or crickets and represent environmental contamination. These compounds include 2,6-bis(1,1dimethylethyl)-4-(1-methylpropyl)-phenol, 6,6-dimethyl-2-methylene-(1S)-bicyclo[3.1.1]heptane, 3-ethyl-2,5-dimethyl-pyrazine, (E)-2-octenal, (1R)-2,6,6-trimethylbicyclo[3.1.1]hept-2-ene, and decanal. All these listed compounds were present in the open egg or cricket samples; therefore, it can be assumed that these were a product of environmental contamination. Regarding the overall persistence of each specimen type, both the SLF eggs and crickets depicted a weekly change in the reported VOC profile. PCA analysis corroborated that the primary odor set of compounds did not cluster as a function of the week, indicating continuous flux over time. The exceptions can be seen in the open SLF egg and cricket samples as weeks 1 through 4 of the eggs are clustered, and weeks 3 and 4 of the crickets are clustered. This would indicate that between these weeks, the VOC profile of each specimen type remained stable. The other exception can be seen in the closed SLF egg samples as weeks 0 through 3 and 5 are clustered, indicating the VOC profile in between these weeks remained stable in the SLF eggs only. The VOC abundance of the crickets, both open and closed, held somewhat until week 3 when a steep decline was seen. The VOC abundance of the eggs, both open and closed, was relatively low throughout the 6-week period. However, there was an increase in VOC abundance in the closed eggs in week 3, and this trend persisted until the last week of the sampling period. It was also at week 3 where the VOC abundance of the closed eggs, open crickets, and closed crickets began to equal out, and this trend also persisted until the last week of the sampling period.

This trend was also depicted in the one-way ANOVA measuring the effect of dissipation type (closed or open lid) on VOC accumulation. In the SLF egg samples, a *p*-value less than 0.05 was produced (*p* = 0.0005), indicating a statistically significant effect on VOC accumulation as a function of lid type. This analysis reveals that it is more beneficial for the SLF egg samples to be stored with a closed-lid vial containment system between sampling times, as leaving the vials open would significantly affect the VOC accumulation detected in the samples. For the cricket samples, the one-way ANOVA test was run to analyze the VOC accumulation in relation to lid type, as well. In the cricket samples, a *p*-value slightly greater than 0.05 was produced (*p* = 0.0574); therefore, it was not statistically significant for the analysis of VOC accumulation in relation to lid type. Given the tight statistical threshold, it can also be said that lid type did affect the cricket samples as well, and, given the higher instrumental response on VOC abundance, might have caused the slight increase in *p*-value. Using these results, it is recommended that any future sampling of SLF eggs or training aids be kept closed between sampling times and use only cold-killed eggs as a treatment method for collected samples. While cricket VOC accumulation was slightly affected by lid condition, the integrity of the volatile odor array pattern is better safeguarded by having samples closed to prevent the introduction of extraneous environmental volatile contaminants.

## 5. Conclusions

SLF eggs, cricket, and bark samples were analyzed using headspace SPME extraction and then further analyzed using a GC-MS. The major conclusion that this study presents is that SLF eggs have a distinctive chemical odor profile comprised of the following six primary odor compounds: anisole, tridecane, dodecane, 2-nonanone, 3-octanone, and D-limonene, as these were the most frequently seen in the various samples. The SLF egg and cricket samples did not share many compounds, thereby showing a distinctive odor profile across insect species. The SLF egg and bark samples shared one compound, anisole, and this would be expected because the SLF eggs were laid and collected from the bark samples provided. After 6 weeks of frozen storage, there was still detection of odor volatiles; however, the VOC profile of the SLF eggs and crickets was not reproducible in terms of volatile odor ratios. Further research with greater sample sizes and an increased number of replicates may enhance our understanding. For the room temperature study as a function of lid type, the VOC profile of the SLF eggs and crickets did not persist consistently across the 6 weeks, with the introduction of extraneous volatiles in the open containment vials and a significant effect on volatile accumulation depletion with the open vial SLF egg samples.

A recommendation for future studies would be to expand the use of distractor odors, whether that be more insect species used or other types of host plants, to ensure that the training aid for SLF eggs is indicative of the SLF itself. This study provides the first odor profiling from *Lycorma delicatula* egg sample masses yielding SPME-GC/MS as a viable technique for volatile analysis. While additional research is needed, the results presented provide a foundation to support analysis-driven decisions in the development of best practices for current SLF canine detection eradication efforts of this invasive pest in the United States.

## Figures and Tables

**Table 1:** Weights of the SLF egg and distractor samples used (*n* = 6 per sample type).

		Weight (g)
Eggs	Sample 1	0.4716
	Sample 2	0.5773
	Sample 3	0.5606
	Sample 4	0.4957
	Sample 5	0.5057
	Sample 6	0.5242
	Average: SE:	0.5225 ±0.0163
Crickets	Sample 1	0.3332
	Sample 2	0.3150
	Sample 3	0.3311
	Sample 4	0.3860
	Sample 5	0.2667
	Sample 6	0.3342
	Average: SE:	0.3277 ±0.0157

**Table 2:** Weights of the open and closed SLF egg and cricket samples used.

		Weight (g)
Eggs in open vial	Sample 1	0.5836
	Sample 2	0.4991
	Sample 3	0.4266
	Sample 4	0.4411
	Sample 5	0.3343
	Sample 6	0.5978
	Average: SE:	0.4804 ±0.0410
Crickets in open vial	Sample 1	0.2116
	Sample 2	0.1685
	Sample 3	0.1830
	Sample 4	0.3736
	Sample 5	0.2432
	Sample 6	0.3211
	Average: SE:	0.2501 ±0.0331
Eggs in closed vial	Sample 1	0.5393
	Sample 2	0.5796
	Sample 3	0.5378
	Sample 4	0.5690
	Sample 5	0.8026
	Sample 6	0.6467
	Average: SE:	0.6125 ±0.0413
Crickets in closed vial	Sample 1	0.2460
	Sample 2	0.1334
	Sample 3	0.2252
	Sample 4	0.3830
	Sample 5	0.3683
	Sample 6	0.2771
	Average: SE:	0.2721 ± 0.0381

**Table 3:** Weights of the bark samples used.

		Weight (g)
Bark	Sample 1	0.2623
	Sample 2	0.3352
	Sample 3	0.6344
	Sample 4	0.5824
	Sample 5	0.5307
	Sample 6	0.4100
	Sample 7	0.2013
	Sample 8	0.2346
	Sample 9	0.2432
	Sample 10	0.2376
	Sample 11	0.2483
	Sample 12	0.2443
	Average: SE:	0.3470 ± 0.0443

## Data Availability

The original contributions presented in the study are included in the article; further inquiries can be directed to the corresponding author.
